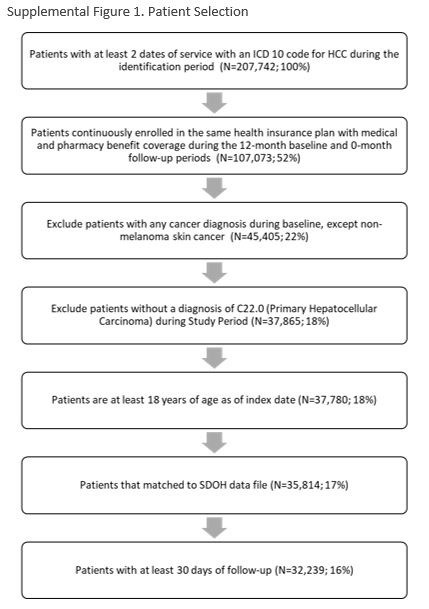# Impact of social determinants of health on hepatocellular carcinoma surveillance, treatment, and health care costs

**DOI:** 10.1097/HC9.0000000000000517

**Published:** 2024-10-10

**Authors:** Amit G. Singal, Karl M. Kilgore, Elizabet Shvets, Neehar D. Parikh, Neil Mehta, A. Burak Ozbay, Christie Teigland, Omar Hafez, Amy Schroeder, Audrey Yang, Jill Schinkel

**Affiliations:** 1 Division of Digestive and Liver Disease, UT Southwestern Medical Center, Dallas, Texas, USA; 2 Inovalon Inc., Bowie, Maryland, USA; 3 Avalere Health, Washington, District of Columbia, USA; 4 Department of Internal Medicine, Division of Gastroenterology and Hepatology, University of Michigan Medical School, Ann Arbor, Michigan, USA; 5 Department of Medicine, University of California San Francisco, San Francisco, California, USA; 6 Exact Sciences Corporation, Madison, Wisconsin, USA

**Keywords:** Liver cancer, health disparities, socioeconomic status, screening, treatment, healthcare costs

## Abstract

**Background:**

The impact of clinical factors and social determinants of health on treatment patterns and health care costs among patients with HCC is unknown.

**Methods:**

Using 100% Medicare Fee-For-Service claims and a commercial multipayor claims database, we identified patients diagnosed with HCC from January 1, 2017, to December 31, 2020. Surveillance receipt was defined 12 months prior to HCC diagnosis, whereas treatment and health care costs were assessed post-HCC diagnosis. Multinomial logistic regression was used to assess the association between demographics, social determinants of health, and surveillance or HCC treatment. Multivariable generalized linear regression was used to identify factors associated with total health care costs.

**Results:**

Of the 32,239 patients with HCC (mean age 68 y, 67% male, 73% White), 70% received surveillance and only half (51%) received any treatment. Curative treatment receipt was higher among those with prior surveillance (24% with CT/MRI and 18% with ultrasound vs. 9% with no surveillance). Curative treatment was independently associated with HCC surveillance and inversely associated with Black race, lower education level, and diagnosis in the year 2020 (COVID-19 year). Higher health care costs were independently associated with Black race, low English proficiency, living alone, and diagnosis in 2018–2020, and inversely associated with CT/MRI-based surveillance.

**Conclusions:**

Race and social determinants of health were independently associated with curative treatment receipt and health care costs. Increasing access to high-quality HCC surveillance may improve treatment receipt and reduce health disparities among patients with HCC.

## INTRODUCTION

HCC is the fifth leading cause of cancer-related deaths in the United States, with an estimated 29,380 deaths in 2023.^1^,^2^ Although HCC survival has improved over time, the 5-year survival rate remains <25%. One of the strongest predictors of HCC prognosis is tumor stage at diagnosis, with curative treatments available for early-stage HCC, while more advanced-stage tumors are generally treated with palliative therapies.^3^

While HCC affects all populations, several sociodemographic characteristics, including age, sex, race/ethnicity, and socioeconomic status, have been associated with an increased HCC burden. Patients from socioeconomically disadvantaged populations and racial/ethnic minorities experience higher HCC incidence and mortality rates.^4^ Additionally, men are disproportionately affected by HCC, with incidence and mortality rates over double those of females.^4^,^5^,^6^,^7^ Finally, Black patients are also more likely to have HCC diagnosed at a later disease stage, less likely to receive curative treatments than White, Hispanic, and Asian patients, and exhibit shorter survival.^8^, Rich 2019,^9^,^10^,^11^,^12^ Disparities in health care are often impacted by social determinants of health (SDOH), such as household income, health care literacy, medical mistrust, and financial barriers.^13^,^14^ Provider-level and system-level factors such as implicit bias and access to multidisciplinary care can also contribute to health disparities.^15^,^16^ These factors have been associated with failures along the cancer care continuum, including underuse of HCC surveillance, diagnostic and therapeutic delays, and underuse of curative treatment among those with early-stage HCC.^17^,^18^,^19^

Currently, most studies have focused on the association between sociodemographic characteristics and HCC cancer care delivery, with few studies examining SDOH. Therefore, the association between SDOH and HCC surveillance patterns, health care utilization, and costs is not well understood. The goal of this paper is to assess the impact of SDOH factors on surveillance and treatment receipt after controlling for etiologies and other risk factors that might impact treatment and surveillance among a large cohort of patients with HCC in a real-world setting.

## METHODS

### Study design and data sources

This retrospective claim-based cohort study used 100% Medicare Fee-for-Service (FFS) data (Parts A, B, and D) and Medicare Advantage, commercial, and managed Medicaid claims from Inovalon’s Medical Outcomes Research for Effectiveness and Economics Registry.^20^ The payer-sourced data constitute a warehouse of health care administrative claims from more than 150 US health plans operating in all 50 states. The FFS and payer-sourced data included complete enrollment and claims histories across all health care settings and service types.

Data on SDOH were sourced from Acxiom Market Indices data, which is an aggregation of data drawn from multiple individual and household databases at the 9-digit ZIP code level.^21^ Prior studies have demonstrated a close association between the social and economic characteristics of individuals’ neighborhoods and their health behaviors and outcomes.^22^,^23^ Given available data, a neighborhood in our study is defined as households within a 9-digit ZIP code level, which includes about 5 households on average. Because data on SDOH variables were not available for each individual patient but only at the 9-digit zip code level, patients in claims were assigned the SDOH characteristics of their corresponding ZIP code via a secure tokenization process. Rurality was also assigned based on patient ZIP code using US Department of Agriculture rural-urban commuting areas^24^ and coded as urban, suburban, large rural town, and small town/isolated rural. This retrospective study involved only secondary analysis of existing, de-identified datasets, and therefore does not constitute human subjects research as defined at 45 CFR 46.102.

### Study population

Patients newly diagnosed with primary HCC between January 1, 2017, and December 31, 2020, were included. To establish index date (initial HCC diagnosis), we used the presence of at least 2 claims on different dates with ICD-10-CM codes C22.0, C22.8, or C22.9. Given diagnosis codes C22.8 and C22.9 are less specific liver cancer codes that may sometimes be used as placeholders until a more specific diagnosis can be made, patients were required to have at least 1 claim for C22.0 during the study period. We included patients who were 18 years of age or above and continuously enrolled in their health plan with medical and pharmacy coverage for at least 12 months prior to the index (baseline period) and at least 1-month postdiagnosis (follow-up period). One month was selected as the minimum duration of follow-up postdiagnosis to provide valid estimates for treatment type and treatment costs. Patients who did not survive at least 30 days following diagnosis were excluded to account for patients being diagnosed with terminal-stage HCC where curative treatment is not warranted. In addition, patients who did not survive at least 30 days following diagnosis had no treatment costs and would attenuate treatment cost estimates toward 0. Patients were also excluded if they had any cancer diagnosis during baseline, except for nonmelanoma skin cancer, or had missing values for any of the SDOH variables defined below.

Recognizing that the high rate of patients with no surveillance or treatment could be related to poor life expectancy (eg, liver dysfunction or medical comorbidity), we performed a sensitivity analysis in which patients were required to remain alive and have continuous coverage for longer minimum follow-up periods (≥3 and ≥6 mo).

### Study variables

Demographic variables measured at baseline included age at index (<40, 40–64, 65–74, 75–79, and ≥80 y), sex, census region (Northeast, Midwest, South, West, unknown), and payer type (Medicare FFS, Medicare Advantage, Commercial, managed Medicaid). Race and ethnicity were categorized as Asian, Black, Hispanic, White, or other. The index year, 2017–2020, was included to identify potential time trends, including the impact of COVID-19.

Clinical characteristics measured over the baseline period included Charlson Comorbidity Index scores (CCI: 0, 1–2, 3–4, 5–6, 7+) well as indicators for the presence^25^,^26^ as well as indicators for the presence of a set of HCC etiology conditions and cirrhosis. Indicators of etiology included alcohol abuse, HBV, HCV, and metabolic dysfunction–associated steatotic liver disease (MASLD). The MASLD indicator included patients with diagnosis codes for MASLD and metabolic dysfunction–associated steatohepatitis or diagnosis codes for type 2 diabetes, obesity, or metabolic syndrome in the absence of codes for alcohol abuse, HBV, or HCV. Cirrhosis was further subcategorized into decompensated and compensated cirrhosis based on the presence of ascites, variceal bleeding, or HE.^27^ Patients may have multiple etiologies. A flag was created for patients without any indicators of etiological factors.

#### SDOH variables

Income was measured as a neighborhood-level variable reflecting household income relative to the federal poverty level (FPL) and categorized as low (<200% FPL), middle (201%–300% FPL), and high (>300% FPL). Utilizing income relative to FPL, rather than median household income, adjusts for both regional costs of living differences and number of people in the household. Other 9-digit ZIP-level SDOH variables included education level (percentage of the population with a high school diploma or less); unemployment rate; percentage owning their own home; percentage living alone; percentage of speaking English not well or not at all; and percentage who do not own their own vehicle.

Surveillance type was assessed over the 12-month period prior to index and categorized hierarchically as (1) CT or MRI (with or without ultrasound [US]), (2) US (but without CT or MRI), (3) Alpha-fetoprotein (AFP) alone (without CT, MRI, or US), and (4) None. CT or MRI done within 3 months prior to index or in the emergency department were not included for determining surveillance type.

The type of HCC treatment was identified during follow-up using ICD-10-PCS procedure, Healthcare Common Procedure Coding System, Common Procedural Terminology, and National Drug Codes.^28^,^29^ Treatment was considered curative if patients received liver transplantation, surgical resection, or local ablation at any time postindex. Patients who received arterial-based or systemic therapies were considered as receiving noncurative treatment. Patients who did not receive HCC-directed therapies were classified under “no treatment.”

Total health care costs were calculated as the total all-cause allowed (paid) amounts in claims, assessed over the follow-up period (index date until health plan disenrollment or the end of the study period), reported in dollars, inflation-adjusted to 2020 using the Medical Care Component of the Consumer Price Index, and standardized to per-patient-per-month (PPPM) values.

Health care utilization included the number of emergency department visits over the follow-up period and was reported as the number of visits per 1000 patients per month (P1000PPM).

### Statistical analysis

Descriptive statistics, including means, SD, and medians for continuous data and frequencies and percentages for categorical data, are reported for baseline patient characteristics, SDOH variables, surveillance type, treatment type, total health care costs, and emergency department visits. Generalized linear regression modeling analyses were used to assess the relationship between the demographic, clinical, and SDOH variables listed in the variables section above and the 3 primary response variables:

1. Surveillance type, using multinomial logistic regression, was reported as OR and 95% CI. Surveillance was assessed as a categorical variable, with the following categories: CT/MRI, US, AFP, and no surveillance (reference group).
2. Treatment type, using multinomial logistic regression, and reported as OR and 95% CI. Treatment type was assessed as a categorical variable, with the following categories: curative, noncurative, and no treatment (reference group).
3. Total health care costs, using generalized linear regression modeling with log link function and gamma distribution, were reported as cost ratios and 95% CI. Total health care costs were assessed as a continuous variable on a PPPM basis.

For models 2 and 3, the type of surveillance was also included as a possible explanatory variable.

In addition to analyses in the entire study cohort, we conducted descriptive subgroup analyses among those with at least 3 and at least 6 months of follow-up after the index visit. These subgroup analyses were performed to exclude patients with possible poor prognosis in whom HCC surveillance and treatment may not have been warranted.

All analyses were performed using SAS software, version 9.4 for Unix (SAS Institute Inc., Cary, NC).

## RESULTS

### Baseline demographic and clinical characteristics

The study included a total of 32,239 patients diagnosed with HCC (Table 1), of whom 19,841 (73%) were White, 3816 (14%) Black, 2056 (8%) Hispanic, 1628 (6%) Asian, and 4898 (15%) were unknown (Table 1A). The mean age was 68 years, and 21,506 (67%) were male. The distribution of patients across regions was relatively uniform, with 11,543 (36%) residing in the South, 7978 (25%) residing in the West, 6640 (21%) residing in the Northeast, 6059 (19%) residing in the Midwest, and 19 (0.1%) were unknown. Most patients in our sample had Medicare FFS coverage (64%), followed by Managed Medicaid (16%), commercial (12%), and Medicare Advantage (8%).

**TABLE 1:** Baseline patient demographics, clinical characteristics, and social determinants of health

Characteristic	Level	N (%)
Total no. patients	—	32,239
A. Demographic characteristics		
Age	Mean	68.3
	SD	10.7
	Median	68
	Age <40	392 (1)
	Age 40–64	10,493 (33)
	Age 65–74	12,835 (40)
	Age 75–79	4020 (12)
	Age ≥80	4499 (14)
Sex	Male	21,506 (67)
	Female	10,733 (33)
Race/ethnicity	White	19,841 (73)
(N, % of known)	Black	3816 (14)
	Hispanic	2056 (8)
	Asian	1628 (6)
	Unknown	4898 (15)
Census region	Northeast	6640 (21)
	Midwest	6059 (19)
	South	11,543 (36)
	West	7978 (25)
	Other/unknown	19 (0)
Index year	2017	9840 (31)
	2018	8729 (27)
	2019	8,056 (25)
	2020	5,614 (17)
Payer type	Medicare FFS	20,711 (64)
	Medicare advantage	5,085 (16)
	Commercial	3,750 (12)
	Managed Medicaid	2,693 (8)
Total no. patients		32,239
B. Clinical characteristics		
CCI	Mean	4.5
	SD	3.2
	Median	4
	0	2023 (6)
	1–2	8505 (26)
	3–4	6837 (21)
	5–6	6745 (21)
	≥7	8129 (25)
Prior Dx cirrhosis^a^		18,287 (57%)
Decompensated (N, % of cirrhosis)		8852 (48)
Compensated (N, % of cirrhosis)		9435 (52)
Prior Dx alcohol abuse^a^	—	5170 (16)
Prior Dx hepatitis B^a^	—	2037 (6)
Prior Dx hepatitis C^a^	—	10,450 (32)
Prior Dx NAFLD^a^	—	11,737 (36)
None of above^a^	—	3852 (12)
Total no. patients	—	32,239
C. Social determinants of health		
Income	High (>300% FPL)	12,938 (40)
	Middle (201%–300% FPL)	12,634 (39)
	Low (≤200% FPL)	6667 (21)
Rurality	Urban	23,749 (74)
	Suburban	3177 (10%)
	Large rural town	2920 (9%)
	Small town/isolated area	2393 (7%)
Education^b^	% HS diploma or less	— (43)
Unemployment^b^	% unemployed	— (4)
Home own vs. rent^b^	% own	— (66)
Living alone^b^	% single-person households	— (59)
English language^b^	% not well or at all	— (5)
Transportation^b^	% no vehicle ownership	— (10)
Total no. patients		32,239
D. Surveillance type		
Surveillance type	CT/MRI	6318 (20)
	US	13,281 (41)
	AFP	3023 (9)
	None	9617 (30)

^a^ Percents do not sum to 100; patients can fall into multiple categories.

^b^ Mean percent of neighborhood.

Abbreviations: AFP, Alpha-fetoprotein; CCI, Charlson Comorbidity Index; FFS, Fee-for-Service; FPL, federal poverty level; US, ultrasound.

Approximately one-third (31%) of patients in our sample were diagnosed with HCC in 2017, 27% in 2018, 25% in 2019, and 19% in 2020. Overall, patients had a mean CCI score of 4.5, and 25% of patients had a CCI ≥7 (Table 1B). Most patients also had a documented diagnosis of cirrhosis at the time of HCC diagnosis (57%). Of the patients with cirrhosis, half (48%) had decompensated cirrhosis. Roughly one-third (32%) of patients had hepatitis C, 36% had MASLD, 16% had alcohol-associated liver disease, 6% had hepatitis B, and 12% had no known etiology.

Patients primarily lived in urban settings (74%), while only 10% resided in a suburban area, 9% resided in a large rural town, and 7% resided in a small rural town (Table 1C). Most patients were living above the poverty level (40% at >300% of the FPL and 39% at 201%–300% FPL). On average, patients lived in neighborhoods where 43% of people completed high school or less, 59% lived in a single-person household, 66% of people owned their own home, 5% of people spoke English not well or not at all, 10% of people had no vehicle, and average unemployment rate was 4%.

### Model 1: surveillance receipt

Of the patients with ≥1 month of follow-up following HCC diagnosis, 20% had received prior CT and/or MRI, 41% US, 9% AFP, and 30% had received no prior surveillance. In a subgroup analysis among patients with ≥3 months and ≥6 months of follow-up post-index, the proportion of patients without surveillance decreased slightly to 28% and 27%, respectively, whereas CT/MRI-based surveillance increased slightly to 21% and 23%, respectively. US-based surveillance remained consistent at 41% across all subset follow-up periods (Table 2).

**TABLE 2:** Surveillance type and treatment type for primary sample (≥1 mo of follow-up) and for 2 subsets with ≥3 and 6 months of follow-up

	Total patients with ≥ 1 mo of follow-up	Total patients with ≥3 mo of follow-up	Total patients with ≥6 mo of follow-up
Total patients	32,239 (100)	26,622 (100)	21,461 (100)
Surveillance type			
CT/MRI	6318 (20)	5670 (21)	4883 (23)
US	13,281 (41)	10,923 (41)	8697 (41)
AFP	3023 (9)	2545 (10)	2082 (10)
None	9617 (30)	7484 (28)	5799 (27)
Treatment type			
Curative	5324 (17)	5108 (19)	4584 (21)
Noncurative	11,264 (35)	10,427 (39)	8640 (40)
No HCC-related treatment	15,651 (49)	11,087 (42)	8237 (38)

Abbreviations: AFP, Alpha-fetoprotein; US, ultrasound.

**FIGURE 1:**
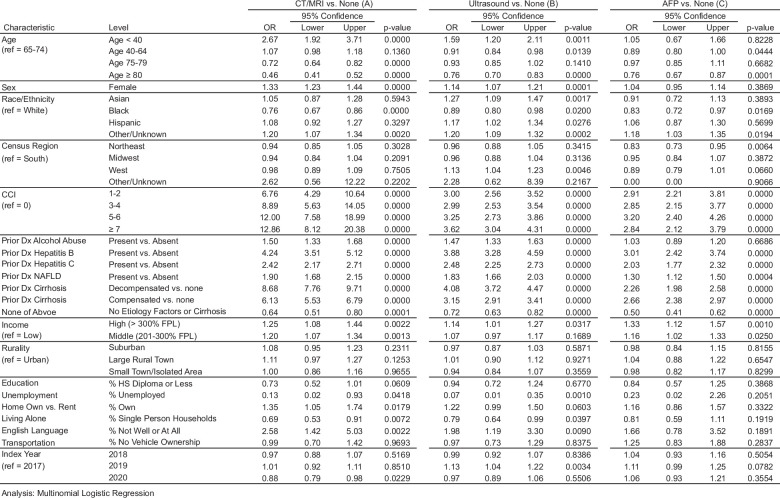
Rates of surveillance (adjusted): OR for surveillance type during baseline (reference group: no surveillance)—all predictors. (A) CT/MRI versus no surveillance. (B) Ultrasound versus no surveillance. (C) AFP versus no surveillance. *Note:* Education, unemployment, home own versus rent, living alone, English language, and transportation are continuous variables that represent % of a neighborhood. A neighborhood in our study is defined at the 9-digit ZIP code level, which includes about 5 households on average. Because data on SDOH variables was not available for each individual patient, but only at the 9-digit zip code level, patients in claims were assigned the SDOH characteristics of their corresponding ZIP code.

In the multinomial logistic regression analysis, factors associated with imaging-based surveillance included younger age, White race, higher CCI, employment, higher income, homeownership, household size, and English proficiency (Figure 1). As anticipated, younger age and other socioeconomic factors related to financial prospects, such as homeownership and income, were positively associated with receipt of surveillance. Complete modeling results for all explanatory and response variables are shown in Figure 1.

Compared to patients aged 65–74 years, younger patients aged below 40 years were more likely to receive surveillance, whereas older patients were less likely to receive surveillance. Asian and Hispanic patients were more likely to receive US than White patients, while Black patients were less likely to receive US than White patients. Patients with higher median neighborhood incomes (>200-300% of the FPL), low English proficiency, and high homeownership had higher odds of receiving US or CT/MRI surveillance. Conversely, patients living in neighborhoods with high unemployment or living alone had lower odds of receiving US or CT/MRI surveillance. Other positively associated predictors for both US and CT/MRI surveillance included female sex and higher comorbidity, whereas lack of documented cirrhosis or liver disease etiology was negatively associated with both types of surveillance.

### Model 2: treatment type

Curative treatment was performed in only 17% of patients, whereas 35% received noncurative treatment, and nearly half of the patients (49%) received no HCC-related treatment (Table 2). In a subgroup analysis of patients with ≥3 months and ≥6 months of follow-up postindex, the proportion of patients without any treatment decreased to 42% and 38%, respectively. Conversely, the proportion of patients who received curative treatment increased to 19% and 21%, respectively.

**FIGURE 2:**
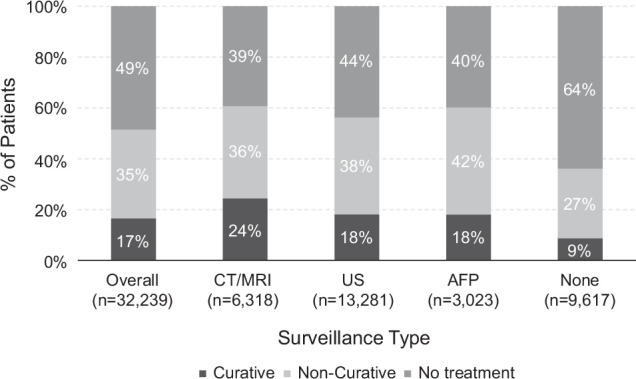
Treatment type, overall, and by surveillance type (unadjusted).

Patients who received any type of surveillance were more likely to receive treatment than patients with no surveillance. Specifically, patients with CT/MRI were 4.79 times more likely to receive curative treatment, and patients with US were 2.91 times more likely to receive curative treatment than patients with no surveillance. Curative treatment was performed in 24% of patients who received CT/MRI surveillance, 18% of patients who received US surveillance, 18% of those who received AFP, and 9% of patients who received no surveillance (Figure 2).

**FIGURE 3:**
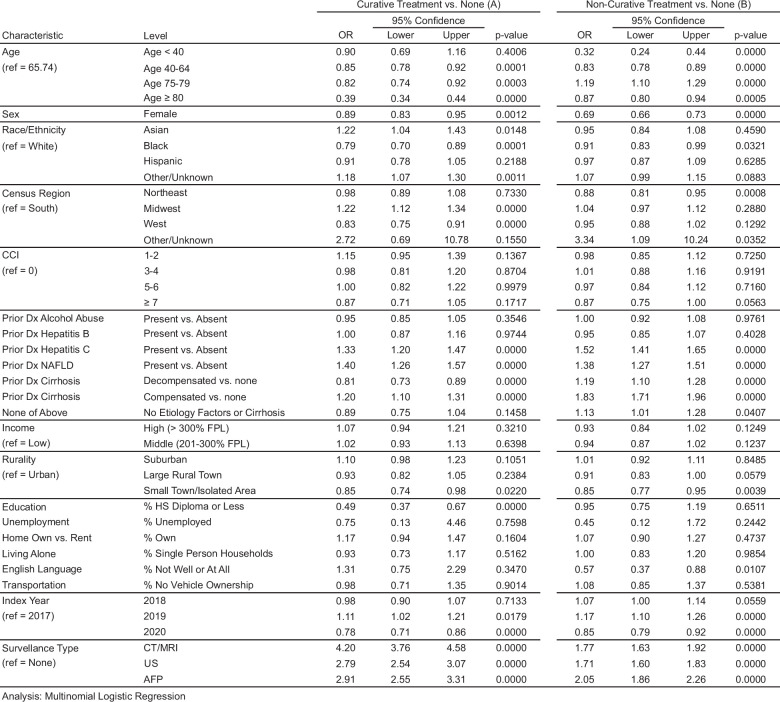
Rates of treatment (adjusted): OR for treatment type during follow-up (reference group: no HCC Treatment)—all predictors. (A) Curative treatment versus no treatment. (B) Non-curative treatment versus no treatment. *Note:* Education, unemployment, home own versus rent, living alone, English language, and transportation are continuous variables that represent % of a neighborhood. A neighborhood in our study is defined at the 9-digit ZIP code level, which includes about 5 households on average. Because data on SDOH variables was not available for each individual patient, but only at the 9-digit zip code level, patients in claims were assigned the SDOH characteristics of their corresponding ZIP code.

Multinomial logistic regression analysis identified factors associated with receipt of any treatment (curative or noncurative), including age, race, rurality, education, English proficiency, and surveillance type (Figure 3). Complete modeling results for all explanatory variables are shown in Figure 3. Patients who were 40–64 or 75 years of age or older were less likely to receive curative treatment than patients ages 65–74. Compared to White patients, Black patients were less likely to receive curative treatment, while Asian patients were more likely to receive curative treatment. Lower neighborhood education attainment and living in a small town were both associated with lower odds of curative treatment. HCV or NAFLD-related liver disease and compensated cirrhosis were positively associated with curative treatment, whereas female sex and decompensated cirrhosis were negatively associated with curative treatment (Figure 3).

### Model 3: health care costs

**FIGURE 4:**
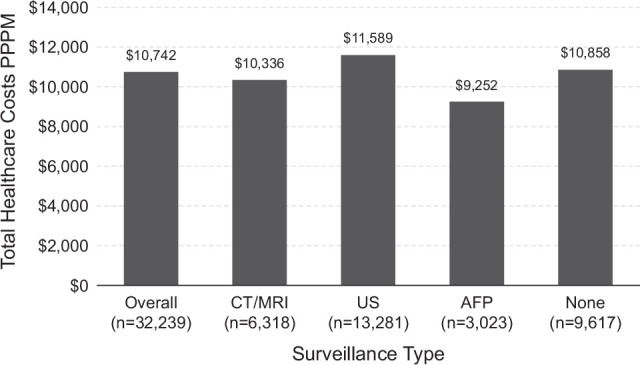
Total health care costs PPPM, overall, and by surveillance type (unadjusted).

Patients had an average of $10,742 total health care costs PPPM (Figure 4). Patients who received CT/MRI surveillance had 25% lower costs than patients with no surveillance, with a $10,336 average cost PPPM for patients who received CT/MRI, $11,589 for patients who received US, $9252 for patients who received AFP, and $10,858 for patients who received no surveillance.

In the multivariable generalized linear regression analysis, factors associated with total health care costs included age, race, CCI, rurality, unemployment, living alone, low English proficiency, access to transportation, and surveillance type. Complete cost modeling results for all explanatory variables are shown in Supplemental Table S1, http://links.lww.com/HC9/B18. The presence of decompensated cirrhosis and increasing comorbidity burden were significantly associated with higher costs, but extremes of age (<40 and ≥80 y), female sex, and compensated cirrhosis were negatively associated with health care costs. Black patients also had 12% greater costs than White patients did. Patients living in a suburban area or large rural town had 8% lower total health care costs PPPM than patients living in an urban area. In addition, living in an area with high unemployment rates was associated with 320% greater costs, living alone was associated with 10% greater costs, low English proficiency was associated with 44% greater costs, and owning a vehicle was associated with 12% greater costs.

## DISCUSSION

In this large national cohort of patients diagnosed with HCC, our findings integrate and complement findings from prior research and further document the important role that race/ethnicity and SDOH play over the entire disease course of HCC. We highlighted the continued underuse of HCC surveillance and treatment in clinical practice, contributing to the continued rise in HCC-related mortality in the United States and worldwide. Several SDOH, including socioeconomic status and educational attainment, make independent contributions to variations in surveillance receipt, HCC treatments, and health care costs.

One of the take-home messages from our analysis is that many patients in our study failed to receive HCC surveillance and curative treatment; less than two-thirds of patients had any imaging in the year prior to HCC diagnosis, and <1 in 5 underwent curative therapy. The underuse of surveillance was associated with several important downstream outcomes, including lower curative treatment and higher costs. These data add to existing studies reporting surveillance benefits and highlight the importance of interventions to increase HCC surveillance. Several studies have shown the benefit of inreach and outreach interventions to increase HCC surveillance, and the American Association for the Study of Liver Disease (AASLD) has called for increased efforts to implement such interventions in routine practice.^30^,^31^,^32^ Outside of these interventions, there have been increased efforts to advance alternative imaging-based or blood-based surveillance strategies, which may have increased patient adoption and improved utilization.^33^,^34^,^35^

In addition to previously reported associations with age, race, ethnicity, liver disease etiology, and severity, we found that several SDOH factors were associated with variation in surveillance receipt.^17^ Specifically, high-income patients were significantly more likely to receive surveillance than low-income patients. These data also highlight the importance of examining the intersectionality of race/ethnicity and socioeconomic status. A NIH Surveillance, Epidemiology, and End Results Program-Medicare analysis highlighted that Black-White disparities were most evident in low socioeconomic status populations and were not observed in the high socioeconomic status group. Imaging-based surveillance has also been associated with educational attainment, employment, homeownership, living situation, and English proficiency.^11^ The finding for English language proficiency is counterintuitive but may be related to higher surveillance among Asian and Hispanic patients, who may be less likely to speak English than their counterparts. The other observed SDOH may contribute to reported surveillance barriers, including transportation, costs of surveillance testing and opportunity costs, and difficulty navigating multiple visits across the health care system.^36^,^37^,^38^ A better understanding of the contributing factors is necessary to inform effective interventions to promote a health equity model, particularly given that the underuse of surveillance and treatment occurs in populations who experience the greatest HCC burden. Interventions will likely need to address multilevel factors, including patient-level (eg, medical mistrust and health literacy), provider-level (eg, addressing implicit bias), system-level (eg, access to care), and policy-level (eg, insurance coverage).^39^

Unsurprisingly, patients with compensated cirrhosis had higher odds of HCC treatment, although it is noteworthy that patients with decompensated cirrhosis had lower odds of curative treatment, highlighting the underuse of liver transplantation. Consistent with prior research, we found that Black patients were significantly less likely to receive curative or noncurative treatments than White patients.^40^ Although many SDOH factors were not associated with HCC treatment, our data highlight urban-rural disparities, with lower curative therapy among those in rural areas. These disparities are likely related to several factors, including access to high-volume centers and multidisciplinary care, both of which are associated with improved HCC outcomes. It is possible that increased access to telehealth visits may mitigate these disparities in the future.^16^,^41^

All-cause health care costs were significantly associated with increasing CCI. As HCC etiology shifts from viral hepatitis to increasing proportions with Alcohol-associated liver disease and metabolic dysfunction-associated steatotic liver disease, it is likely that patients will have increasing comorbidity, highlighting the need for increased efforts to adequately address comorbidity.^42^ Financial burden and toxicity have been reported in several cancers, including recent data among patients with HCC.^43^ Patients with HCC may be particularly prone to financial burden given the concurrent costs of cancer, underlying cirrhosis, and comorbid conditions. We found that higher costs were associated with unemployment, living alone, and poor English proficiency, suggesting that the financial burden is disproportionately impacting populations that can least afford it.

### Limitations

Although our study used a large, diverse multipayer database, medical claims do not capture clinically relevant details which would impact health care utilization and costs. Claims are not able to capture variables such as disease stage, tumor characteristics, liver function, and response to treatment, which may lead to misclassification and unmeasured confounding. Tumor stage and liver dysfunction are both factors that can impact eligibility for surveillance and curative treatment, as well as impact health care costs.

Second, although we normalized costs to per patient per month values to account for variable follow-up periods, all patients were required to have at least 1 month of continuous enrollment to capture outcomes. Patients who died within the first 1 month of diagnosis were excluded from the study, resulting in potential immortal time bias. Adding <1 month of follow-up as an exclusion criterion reduced total N from 35,814 to 32,239 (Supplemental Figure S1, http://links.lww.com/HC9/B19), meaning this criterion only reduced N by 3575 (10%). Given that the majority of patients had more than 1 month of follow-up (ie, lived longer than 1 mo), our results are still relevant for the majority of patients with HCC. In addition, excluding patients that did not survive at least 30 days following diagnosis can reduce bias and allow for valid estimates of treatment type and treatment costs. Patients who did not survive at least 30 days following diagnosis may have been diagnosed with terminal-stage HCC, where curative treatment is not warranted. In addition, patients who did not survive at least 30 days following diagnosis had no treatment costs and would attenuate treatment cost estimates toward 0.

Finally, to match patients to SDOH, patients were required to have a valid address in their insurance enrollment records. This implies that all patients had insurance and a home. The results of this study may not be generalizable to insecure housing and to uninsured patients.

## CONCLUSIONS

HCC surveillance and curative treatment are underused in clinical practice. Disparities in surveillance and treatment highlight the need for strategies to promote health equity.

## Supplementary Material

**Figure:**
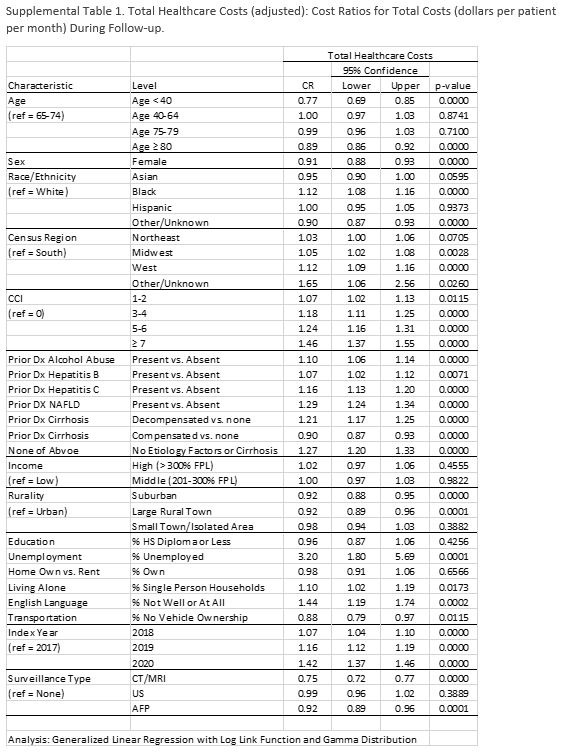


**Figure:**